# Galanin Transgenic Mice with Elevated Circulating Galanin Levels Alleviate Demyelination in a Cuprizone-Induced MS Mouse Model

**DOI:** 10.1371/journal.pone.0033901

**Published:** 2012-03-19

**Authors:** Lin Zhang, Wu Yu, Ingo Schroedter, Jiming Kong, Maria Vrontakis

**Affiliations:** 1 Department of Human Anatomy & Cell Science, University of Manitoba, Winnipeg, Manitoba, Canada; 2 Diagnostic Services of Manitoba Inc. (DSM Inc.) Health Sciences Site, Winnipeg, Manitoba, Canada; Innsbruck Medical University, Austria

## Abstract

Multiple Sclerosis (MS) is a demyelinating autoimmune disease of the central nervous system (CNS) with a presumed autoimmune etiology. Approved treatments for MS are immunoregulatory and are able to reduce the inflammatory components of the disease. However, these treatments do not suppress progressive clinical disability. Approaches that directly protect myelin-producing oligodendrocytes and enhance remyelination are likely to improve long-term outcomes and reduce the rate of axonal damage. Galanin (GAL) is a bioactive neuropeptide that is widely distributed throughout the nervous system and has diverse neuromodulatory effects. In this study, using the cuprizone (CPZ) demyelination model of MS, we demonstrate that GAL has pronounced neuroprotective effects with respect to demyelination and remyelination. Using our GAL transgenic mouse (GAL-Tg), we identified a novel attenuation of OLs against CPZ induced demyelination, which was exerted independently of progenitor cells. Alleviation of myelin breakdown in the GAL-Tg mice was observed to be significant. Furthermore, we observed changes in the expression of the GAL receptor GalR1 during the demyelination and remyelination processes. Our data strongly indicate that GAL has the capacity to influence the outcome of primary insults that directly target OLs, as opposed to cases where immune activation is the primary pathogenic event. Taken together, these results suggest that GAL is a promising next-generation target for the treatment of MS.

## Introduction

Multiple Sclerosis (MS) is an inflammatory demyelinating disease of the brain and spinal cord with a presumed autoimmune etiology. The pathology of MS features focal areas of inflammatory infiltration and demyelination in which oligodendrocytes (OLs) – the myelinating cells of the central nervous system (CNS) – are depleted [1]. During the early stages of MS, some repair of this damage is still possible, likely due to partial remyelination [2] initiated by surviving or newly formed OLs (generated from progenitor cells) [3]. In the primary and secondary progressive forms of MS, a high degree of cortical demyelination has been observed [4], [5], but active cortical lesions show only mild lymphocytic infiltrates [6]. All approved treatments for MS are immunoregulatory, which are able to reduce the inflammatory component of the disease. Unfortunately, these treatments are not effective against progressive clinical disability. However, new treatments that target OLs and myelin sheaths have come under serious consideration [7], [8]. Approaches that directly protect myelin-producing OLs and enhance remyelination may improve long-term outcomes and reduce the rate of axonal damage in MS patients.

Galanin (GAL) is a 29-amino-acid neuroendocrine peptide that is widely distributed throughout the rat, mouse and human CNS, where it functions alongside more “classical” neurotransmitters [9], [10]. It has been demonstrated that GAL acts as a survival and growth-promoting factor for different types of neurons in the peripheral nervous system (PNS) and the CNS [11]. GAL has also been implicated in the control of neurogenesis (i.e., the proliferation, differentiation and/or migration of neural stem cells) in both normal and injured brains [12], [13], [14], [15]. High levels of GAL and GAL receptors (GalR1 and GalR2) are expressed in the subventricular zone (SVZ), the rostral migratory stream (RMS), the subgranular zone of the dentate gyrus (SGZ) and in oligodendrocyte precursor cells (OPCs) of the corpus callosum [12], [13], [16], [17], [18]. Recently, it has been demonstrated that GAL is markedly upregulated in MS lesions including shadow plaques in post-mortem brain tissue from chronic MS sufferers exclusively in microglia [19], although not all microglia were galanin positive. In the same study, GAL was also uperegulated in the CNS of mice with acute inflammation in the EAE model, although here it was exclusively in oligodendrocytes.

One of the best characterized demyelinating mouse models is that of C57BL/6J mice with cuprizone (CPZ), a copper chelator, added to their diet [20]. CPZ, when given in small doses, acts as a neurotoxin that specifically induces the death of OLs and causes inflammation in the resulting demyelinated areas; these effects are reversible, and the removal of CPZ from the mouse feed permits remyelination. The lesions that occur in the brains of CPZ-treated mice are similar to type III and type IV lesions observed in MS [21]. In this mouse model, focal inflammation and demyelination occurs in the brain, however, T lymphocytes do not appear to play a role in these processes [20].

We have previously reported the creation of a GAL-Tg mouse in which GAL is over-expressed in the anterior pituitary gland causing circulating GAL levels to be chronically elevated [22], [23], and to a much lesser extent prolactin (PRL) and growth hormone (GH). In the present study, using the CPZ-mediated demyelination model of MS, we demonstrate that GAL has pronounced neuroprotective effects with respect to demyelination and remyelination and that it directly prevents OL death. Moreover, we also found changes in the expression of the GAL receptor GalR1 during the demyelination and remyelination processes. These results suggest that GAL and/or GAL receptors could represent next-generation therapeutic targets for the treatment of MS.

## Results

### Effects of the over-expression of GAL on body weight

**Figure 1:**
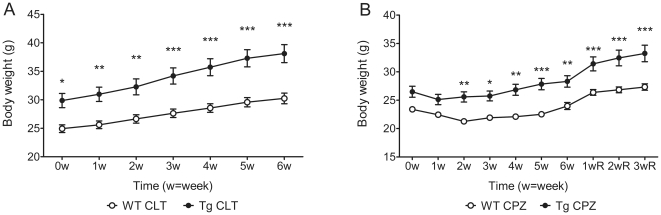
The effect of GAL over-expression on body weight. Body weight was measured at the age of 8–9 weeks old as the starting point 0 w. The raw body weight growth in normal control groups (A) and the CPZ-treated groups (B) is shown. Both the cuprozone groups and the cuprizone-challenge plus 3-week recovery groups were measured at the same time. Data are expressed as the mean ± SEM. (n = 12–17 per group). * p<0.05, ** p<0.01, *** p<0.001.

Initially (at 8–9 weeks of age) Gal-Tg mice were heavier than WT mice, with average body weights of 29.9 ± 4.3 g and 24.9 ± 2.4 g, respectively (mean ± SD).By six weeks later, Gal-Tg and WT mice had gained 27.6±18.3% and 21.3±13.5%, respectively, of their body weight compared to their initial weight(Fig. 1A). In comparison, after treatment with CPZ diets, both Gal-Tg and WT mice lost a significant amount of body weight in the first week. Tg mice lost approximately 5% of their weight after one week, while WT mice lost approximately 10% of their weight after two weeks. However, Tg mice started to gain weight after one week on the diet, while WT mice continued losing weight until the third week (Fig. 1B). Following withdrawal of the CPZ challenge, mice of both genotypes gained weight immediately (but at different rates) (Fig. 1B). There was a statistically significant difference in the rate of body-weight increase between Gal-Tg and WT mice. Two-way mixed-measures ANOVA revealed a significant interaction (F(6,138) = 9.83, p<0.0001) between normal body-weight growth rate and genotype, indicating that the rate of body-weight growth was dependent on genotype. Another two-way mixed-measures ANOVA analysis revealed that, during CPZ challenge, there was a significant influence of genotype on changes in body weight.

### CPZ-induced demyelination in MBP staining is attenuated in GAL-Tg mice

**Figure 2:**
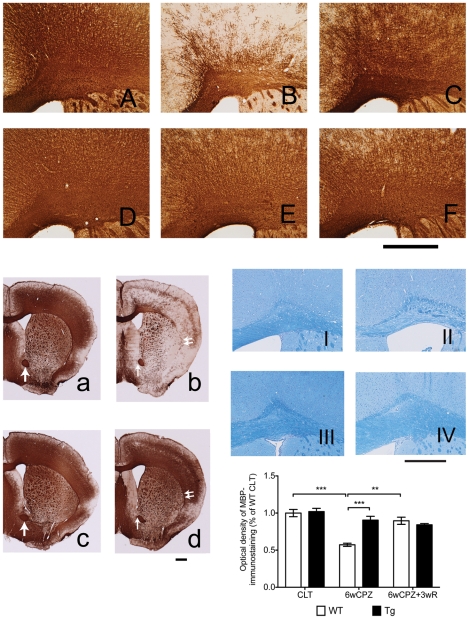
Over-expression of GAL blocks CPZ-induced myelin breakdown. (A)–(F) are photographs of the cerebral cortex and the CC, while (a)–(d) are the photographs of whole brain. Both Tg and WT mice were given 0.3% CPZ for six weeks (6wCPZ), and then the two groups of mice were allowed to recover for three weeks on a normal food diet (6wCPZ+3wR). Mice brains were processed for IHC staining using an antibody against MBP. Consistent with previous studies, we found extreme demyelination in WT mice after six weeks of CPZ challenge: compare (B) and (b) with the control groups (A) and (a). However, MBP staining of Tg mice brains, (E) and (d), indicated that myelin breakdown was not significantly different compared with controls, (D) and (c). After three weeks on a normal diet, the WT mice recovered well (as expected) (C). To verify the IHC staining results, we also used luxol fast blue staining on WT CLT (I), WT 6wCPZ (II), Tg CLT (III) and Tg 6wCPZ (IV) samples (6 µm paraffin sections). The bar graphs represent the measurements of optical density of MBP IHC staining in the cerebral cortex and the CC areas. Arrows show the aca area that was used for color-intensity standardization. Data are expressed as mean ± s.e.m values. (n = 3–5 per group). ** p<0.01, *** p<0.001.

Consistent with previous studies [20], [24], our findings revealed that CPZ challenge caused significant loss of myelin in WT mice, particularly in the CC and in the junction area between the CC and the cerebral cortex (Fig. 2B, Fig. 2b). However, this myelin breakdown was significantly reduced in Gal-Tg mice (Fig. 2E, Fig. 2d). In Gal-Tg mice, myelin degradation in the external capsule of the CC (as demonstrated by the doubled arrow in the whole brain pictures of Fig. 2b, Fig. 2d) were obviously reduced compared with WT mice. In WT mice, after six weeks of CPZ-induced challenge, only a few myelin fibers remained, which were restricted to layer four of the cortex. On the other hand, both WT and Gal-Tg mice from the recovery groups showed dense MBP staining (Fig. 2C, 2F). A two-way ANOVA analysis revealed that genotype significantly affected MPB staining in the control, demyelination and remyelination groups (F(2,16) = 9.56, p = 0.0019). Post-hoc comparison tests found that Gal-Tg mice had a significantly greater level of MBP staining (based on the optical density values) after six weeks of the CPZ challenge. One-factor ANOVA tests on the WT mouse data revealed that the CPZ-induced challenge significantly affected the myelin staining results (F(2,6) = 28.86, p<0.001). We also used Luxol Fast Blue (LFB) staining on normal and CPZ-challenged animals to verify the IHC results, which showed that the integrity of the myelin fibers was similar to that observed in the IHC staining (Fig. 2, images I–IV). Western blotting analysis of MBP protein level changes were consistent with the changes in MBP staining (data not shown).

### Increased circulating GAL alleviates demyelination-related pathogenesis

**Figure 3:**
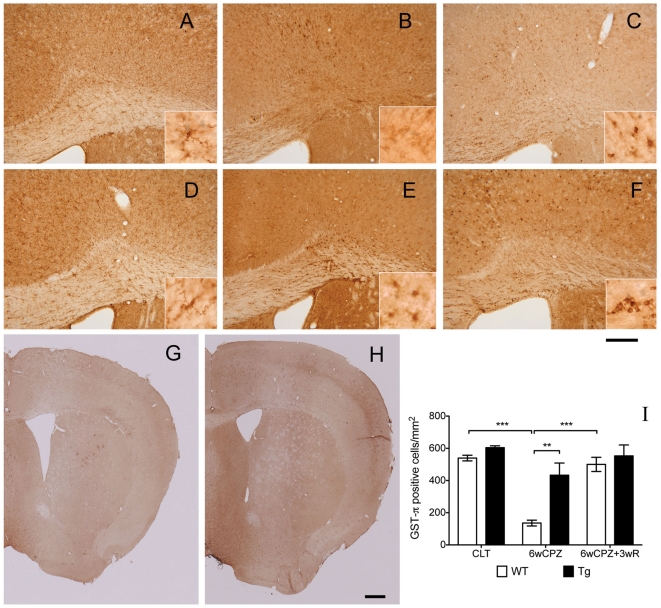
Increased levels of galanin attenuated CPZ-induced oligodendrocyte loss. Mature oligodendrocytes were detected with IHC using a GST-π antibody. Photographs (A–F) were taken from the knee region of the CC, and (G) and (H) are examples of the full-size pictures taken of WT and Tg brains from the 6wCPZ group. The three CC images in the upper panels (A–C) show the WT mice from the CTL, 6wCPZ and 6wCPZ+3wR groups, and the three images in the middle panels (D–F) show the Tg mice from the same groups. In (A–F), high-magnification micrographs were also taken of the CC area using an oil-immersion lens, as shown in the inserts. (I) A bar chart displaying the numbers of GST-π positive cells, which were counted manually. **p<0.01, ***p<0.001, n = 3 in each group. The longer scale bar represents a length of 200 µm and the shorte scale bar represents 500 µm.

The loss of mature OLs was attenuated in GAL-Tg mice. We first examined the mature OLs that survived the CPZ-induced challenge. IHC staining demonstrated that, in control animals, there were widely distributed GST-π positive cells in the cortex (data not shown) and linearly distributed cells in the CC (Fig. 3A, 3D). As expected, after six weeks of CPZ-induced challenge in WT mice, only a few weakly positive cells remained in the CC, and a small number of positive cells remained in the cortical areas (restricted to cortex layer four). However, in Gal-Tg mice, there were still many positive cells after six weeks of CPZ-induced challenge (Fig. 3E, 3H). Furthermore, many positive cells could be observed in WT mice from the recovery group, while no obvious differences were observed in Gal-Tg mice between the demyelination and recovery groups (Fig. 3E, 3F). A two-way ANOVA test on the cell numbers revealed that genotype significantly influenced the number of GST-π-positive cells under the control, demyelination and recovery conditions (F(2,12) = 4.40, p = 0.0370). Post-hoc comparison tests found a significant difference between WT and Tg mice from the 6wCPZ group (p<0.01). A one-way ANOVA test on the WT groups showed that the mean number of positive cells was significantly different across the groups, and Tukey's test revealed significant differences between the WT CLT versus the WT6wCPZ groups (p<0.001) and the WT 6wCPZ versus the WT 6wCPZ+3wR groups (p<0.001). However, a one-way ANOVA test revealed no significant difference among the Tg groups, although the results showed a trend indicating impairment after the CPZ-induced challenge.

### Proliferation and accumulation of OPCs were low in the Gal-Tg mice after CPZ-induced challenge

**Figure 4:**
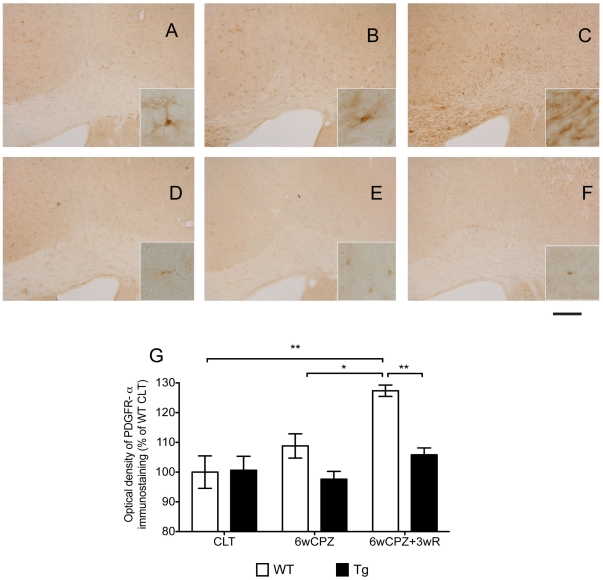
Increased number of PDGFR-α positive cells in WT mice that underwent the CPZ-induced demyelination challenge. WT and Tg mice were given CPZ challenge for six weeks (6wCPZ, B and E) while the control mice (CLT, A and D) received normal CPZ-free rodent chow. After six weeks of challenge, two groups of animals (6wCPZ+3wR, C and F) were allowed to recover for three weeks on a normal CPZ-free diet. The bar graph shows the results of the optical-density measurements of the PDGFR-α-positive cells; a significant difference was observed between the 6wCPZ+3wR WT and Tg groups. The scale bar represents 200 µm in the low-magnification. Data are expressed as mean ± SEM values (n = 3 per group). *p<0.05, ** p<0.01.

The distribution of OPCs was revealed by IHC for PDGFR-α, one of the two commonly used cell markers for OPC identity. Under normal conditions in the control groups, there were few PDGFR-α-positive cells in the brains of WT and Tg mice (Fig. 4A, 4D). After CPZ-challenge, the number of PDGFR-α-positive cells began to increase in the CC of WT mice (Fig. 4B). Furthermore, this positive staining was significantly higher in the brains of WT mice from the recovery group (Fig. 4C) (p<0.05). In contrast with WT mice, the numbers of PDGFR-α-positive cells in Tg mice were similar among the different groups (Fig. 4D–F).

### In GAL-Tg mice the invasion of CPZ-induced reactive astrogliosis was restricted

Without any experimental challenge, most of the GFAP positive cells were limited to the CC area in both WT and Tg mice brains As expected, after the CPZ-induced challenge, increased numbers of GFAP-positive astrocytes could be detected in all regions of the brain in both WT and Tg mice. More precisely, the distribution of the reactive astrocytes in WT mice was diffused through all layers of the cerebral cortex, but in Tg mice, the invasion of reactive astrocytes was limited within layer four of the cerebral cortex, i.e., no labeling was seen in cerebral cortex layers five and six. In the recovery groups, the reactive astrogliosis (in terms of the density of staining) in WT mice was reduced, although the distribution was still dispersed in the cortex, but the diffusion in Tg mice were still restricted in the limited regions.

### The GalR1 receptor is more highly expressed during the demyelination and remyelination (recovery) phases

**Figure 5:**
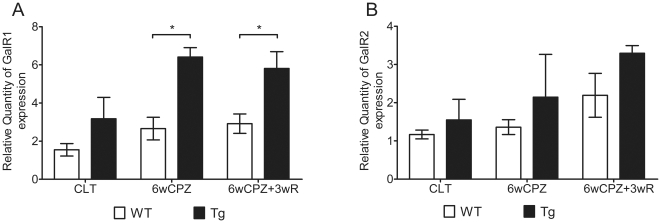
The differential expression of GalR1 and GalR2 in the CC area. RNA samples were extracted from CC areas as demonstrated in Figure 6. The gene expression levels among the groups were normalized to the WT CLT levels (set equal to 1). (A) The expression of GalR1 among the groups. (B) The expression of GalR2 among the groups. Data are expressed as the mean ± SEM values. (n = 3–6 per group). * p<0.05, ** p<0.01.

The expression levels of GAL receptors were normalized to the levels observed in WT mice from the control group. The expression levels of GalR1 were 2.7- and 6.4-fold higher in WT and Tg mice, respectively, in the demyelination groups. The expression levels of GalR1 were increased even further (2.9- and 5.8-fold higher in WT and Tg mice, respectively) in the recovery groups (Fig. 5A). In contrast with GalR1 expression, the expression of GalR2 was increased 1.4- and 2.1-fold in WT and Tg mice, respectively, in the demyelination groups; expression of GalR2 increased 2.2- and 3.3-fold in WT and Tg mice, respectively, in the recovery groups (Fig. 5B).

## Discussion

GAL has diverse biological functions in the nervous system, and the potential for its involvement in myelin development had been demonstrated by Shen et al. [15]. The present study reveals a potential direct effect for GAL in myelin protection against cuprizone induced demyelination.

Unlike other GAL transgenic models, the circulating levels of GAL in our Gal-Tg mice were 10-fold higher than those observed in WT mice [22]. This dramatic increase in circulating GAL provides a unique tool to study its neuromodulatory role. The Blood-Brain Barrier (BBB) was once assumed to be impermeable to peptides. However, it is now widely accepted that peptides are capable of crossing the BBB by both non-saturable and saturable transport mechanism to the extent that they can affect events in the CNS. There exist several potential means whereby circulating GAL could enter the brain. First, the circumventricular organs could provide a broad passageway [25]. Additionally, active transport mechanisms have been described elsewhere, which also protect the substances from rapid degradation [26]. Furthermore, GAL has been shown elsewhere to cross the BBB, as intravenous administration of GAL has quick acting anti-depressant activity and affects sleep EEG recordings [27].

The present study indicates that GAL-receptor signaling plays a key role in modulating CPZ-induced demyelination. Using our GAL over-expressing transgenic mouse model, we identified a novel attenuation of OLs against CPZ-induced demyelination, and we found that this influence was exerted independently of progenitor cells. Alleviation of myelin breakdown in the GAL-Tg mice was observed to be significant.

Myelin basic protein (MBP) is a structural protein of myelin sheath. Furthermore, it is believed to be a vitally important functional protein for myelin assembly and maintenance [28]. MBP IHC staining, which was used in the present study, is a common method for quantitatively measuring myelin sheath levels [29]. Thus, after a six-week exposure to CPZ challenge, the significant loss of MBP staining in WT mice is indicative of severe demyelination, and the attenuation of MBP-staining loss in Tg mice suggests an alleviation of demyelination. The DAB-dye-based IHC staining results were also verified using Luxol Fast Blue staining, another commonly used histological myelin staining method. The loss of MBP protein in WT mice was also demonstrated by western blotting with the same antibody used for the IHC staining (data not shown).

Because the experimental demyelination induced by CPZ is caused by the apoptosis of OLs [20], we next examined the number of mature OLs in the different groups. GST-π is a myelin- and OL-associated enzyme in the brain that is used as a biomarker for mature OLs [30], [31]. For the first time, we show that (with respect to CPZ-induced demyelination) the loss of GST-π positive cells observed in WT mice was greatly alleviated in our GAL- Tg mice. Interestingly, the distribution of protected OLs in the neocortex of the GAL-Tg mice corresponded to the GAL-stained cells in the same area, indicating a direct protective effect of GAL on OL survival. In this study, we also found that the oligodendrocyte precursor cells (OPCs) were highly proliferative and accumulated in response to CPZ-induced oligodendrocyte degradation in the WT mice, in contrast to a minimal response in the Tg mice. It has been suggested that the infiltrating proliferative OPCs are recruited to reverse the loss of mature OLs by proliferation and differentiation and that OL death (in the lesions) is characterized by a repopulation of OPCs prior to new OL formation [32]. These findings support the hypothesis that the lower levels of OPC proliferation and accumulation seen in Tg mice are caused by an improvement in OL survival.

These results provide important insights into the mechanisms by which GAL-receptor signaling influences the phenotypes of central nervous system (CNS) demyelination. Our data strongly indicate that GAL has the capacity to influence the outcome of primary insults that directly target OLs, as opposed to cases where immune activation is the primary pathogenic event. This is an important distinction, as EAE (the principal animal model of central demyelination) is driven by immune cell (principally T cell) activation. Evidence has already been provided to suggest that the influence of GAL in EAE is associated with OLs [19], which is of particular importance given the fact that OL death can be an early event in MS (even preceding immune cell infiltration) [33]. Thus, our study provides evidence for the potential use of GAL as a therapeutic target of oligodendro-cytopathy in MS and compliments the study of Dr. Wynick's group.

One limitation of the study is that in our Gal-Tg mouse besides chronic increase of serum GAL there is an increase, to a much lesser extent, of PRL and GH that they might act as confounders to the observed phenotype. While though both hormones have been described to have an effect on myelination [34], [35], [36], [37], this effect is mainly through the proliferation of OL progenitors (OPS). In our study the most pronounced effect in the Gal-Tg is not the proliferation of OPs, since PDGFR-α-positive cells did not change, but the attenuation of OLs death. We believe that this effect is mainly due to increased GAL levels, although further studies are needed to confirm that. Furthermore, the increased lipid levels of the obese phenotype of our transgenic mice might have also contributed to an accelerated myelination. Studies in progress are defining this issue as well.

In the context of the CPZ model (where the BBB is thought to be intact), the apparent efficacy of increased levels of circulating GAL [38] provides an important insight into the pharmacodynamics of this protein. Our data suggest that (in the context of MS) GAL might be able to protect OLs in regions anatomically distant from the discrete active plaques where the BBB is actually disrupted; this could help eliminate progressive neurodegeneration, independent of the degree of acute inflammatory activity.

It is well accepted that GAL exerts its biological effects through the three G-protein coupled receptors, GalR1, GalR2 and GalR3. These three receptors activate distinct G-proteins that participate in different GalR-signaling pathways, which results in diverse biological functions being activated upon the binding of GAL or other ligands [39]. In this study, a gene expression assay revealed that the expression of GalR1 and GalR2 was differentially altered in the demyelination and remyelination phases, suggesting these two receptors have distinct roles during these two phases. The change in GalR1 expression during the initial challenge phase may be indicative of a protective effect in response to demyelination, while the trend of GalR2 inrease in the later recovery phase, although not significant compared to wild type, may imply the existence of a neurotrophic effect during recovery. Interestingly, the differential involvement of GalR1 and GalR2 in response to a pathological challenge has also be observed in seizure development – the involvement of GalR1 was found to be important during the initiation phase, while GalR2 was found to be more important during seizure maintenance [40]. However, the gene expression assay in this study does not provide direct evidence concerning the mechanisms of GalR1 and GalR2 action in the demyelination and remyelination processes. Further studies are needed to investigate the roles of these two receptors and their downstream effects.

In summary, we have provided evidence that elevated circulating GAL can greatly alleviate CPZ-induced demyelination. Furthermore, we show that the attenuation of demyelination-related impairments (from the effect of elevated GAL) was directly related to a reduction in the death of mature OLs. Gene expression assays also revealed differential responses of GalR1 and GalR2 to demyelination. These findings strongly support the hypothesis that elevated circulating GAL can alleviate CPZ-induced demyelination via GalR1 receptor. Demyelination is the final pathological downstream event in MS, and it is at the core of the diverse symptoms and disabilities caused by this disease. Over the decades, many contributions have been made to the immunoregulatory treatment of MS. However, new strategies for myelin protection are considered to be the best hope for next-generation therapies. Overall, our findings strongly suggest potential therapeutic effects for GAL, with respect to myelin protection, in the treatment MS.

## Materials and Methods

### Experimental animals

All mice, including wild-type (WT; C57BL/6) and homozygous transgenic mice (GAL-Tg) maintained on a C57BL/6, were housed in the University of Manitoba animal facility in a temperature controlled environment (20°C under a 12 h light/dark cycle). Food and drinking water were available ad libitum. Twenty five male WT mice were obtained from Charles River (Montreal, QC, Canada); GAL-Tg mice were generated as previously reported [22], [23] using a 320 bp fragment of the rat growth hormone (GH) promoter fused to the full-length rat preprogalanin cDNA clone. Before the initiation of the experiment, homozygous Gal-T mice were backcrossed to wild type WT; C57BL/6 from Charles River and brought again to homozygosity. Thirty six GAL-Tg male mice were used in this study, to avoid the effects of fluctuating steroid hormones in female mice on the expression of GAL [23], [41]. 3–6 mice were used per group and the whole experiment was repeated twice. All procedures were in accordance with the Animal Protocol Review Board of the University of Manitoba which has approved this study under the protocol #10-013/1 (principal investigator Dr.Vrontakis). Cuprizone-induced demyelination

To induce demyelination, both WT and GAL-Tg mice at 8–9 weeks of age were fed with a diet containing 0.3% (w/w) CPZ (C9012-25G, Sigma-Aldrich) for six weeks. No lethality was observed due to treatment. CPZ powder was well blended into milled LabDiet rodent chow (Prolab® RMH 3000 5P00, 22.0% crude protein, 5.0% crude fat, 5.0% crude fiber, 6.0% ash, and 2.5% added minerals) using a food processor (43-1976-0, KitchenAid), then the blended diet powder was re-pelleted by extrusion through a 30 ml plastic tube. The CPZ diet was freshly prepared and given to the mice twice a week while monitoring for body weight. To allow for remyelination, animals that had received the CPZ diet were put back on a normal diet for an additional three wks. The experimental mice were divided into the following three groups: control (CLT), demyelination (6wCPZ) and remyelination (6wCPZ+3wR). Body weight and general behaviors (grooming, activity, etc.) were monitored twice a week.

### Immunohistochemistry

For the Immunohistochemistry (IHC) analysis, half of animals in each group were anesthetized by isoflurane inhalation, perfused intracardially with 0.1 M Phosphate buffered saline (PBS; pH 7.4) containing 50 U/ml heparin sodium, followed by 4% paraformaldehyde prepared in 0.1 M PBS. The brains were then dissected out and post-fixed in the same fixation solution at 4°C overnight, followed by cryoprotective treatment in 0.1 M PBS containing 30% sucrose at 4°C (until the brains no longer floated). The dehydrated brains were snap-frozen on dry ice and stored at −80°C until sectioning. Serial coronal sections (25 µm thick) of the frozen brains were made using the Leica SM2400 sliding microtome equipped with a freezing plate. The free-floating sections were then kept in 24-well plates containing 0.1 M PBS at 4°C. Sections corresponding to levels 185–195 of the High Resolution Mouse Brain Atlas (Sidman et al.; http://www.hms.harvard.edu/research/brain/atlas.html) were used in this study. Staining was performed using the avidin-biotin-peroxidase complex technique. Procedures were carried out on a slow shaker at room temperature, unless otherwise indicated. Briefly, free-floating sections were washed in 50 mM Tris Buffered Saline (TBS, pH 7.4) three times (5 min each) followed by pre-treatment with 3% hydrogen peroxide for 30 min. Sections were then washed in TBS and incubated for 1 h in a blocking solution consisting of 5% normal goat serum and 0.3% Triton-X100 in TBS. Subsequently, the sections were incubated in the same blocking solution overnight at 4°C with primary antibodies (Table 1). After washing in TBS containing 0.1% Tween-20 (TBST), sections were incubated with biotinylated secondary antibodies (all secondary antibodies were purchased from Vector Laboratories, Ontario, CA) for 1 h, followed by three washes in TBST. Sections were further treated with the peroxidase-coupled avidin-biotin complex (ABC Peroxidase Staining Kit, Cat # 32020, Pierce) for 1 h. Finally, immune-precipitated sections were visualized using the DAB Peroxidase Substrate Kit (3,3′-diaminobenzidine; Cat # sk-4100, Vector Lab). After mounting on the clean pre-coated slides (Superfrost Plus Microscope Slides, Cat # 12-550-15, Fisher), sections were then air-dried on a 37°C slide warmer overnight. The following day, sections were post-treated with a series of increasing concentration alcohols from 70% to 100%, followed by two treatments with xylene. Control slides were processed in parallel with experimental slides using the same agents, except without primary antibodies. No positive staining was observed in the control slides.

**Table 1:** Primary antibodies used for immunohistochemistrical staining.

	Target	Dilution ratio	Catalog #	Supplier
**Anti-MBP (C-16)**	Myelin	1∶1000	Sc-13914	Santa Cruz
**Anti-GST-π**	Mature OL	1∶1000	610719	BD Biosciences
**Anti-GFAP**	Astrocyte	1∶1000	MAB360	Millipore
**Anti-CD140a**	PDGFR-α/OPC	1∶500	558774	BD Biosciences

Abbreviations: MBP – myelin basic protein; GST-π – Glutathione S-Transferase π form; GFAP – glial fibrillary acidic protein; PDGFR-α – alpha-type platelet-derived growth factor receptor; OPC – oligodendrocyte precursor cell; OL – oligodendrocyte.

### RNA preparation

**Figure 6:**
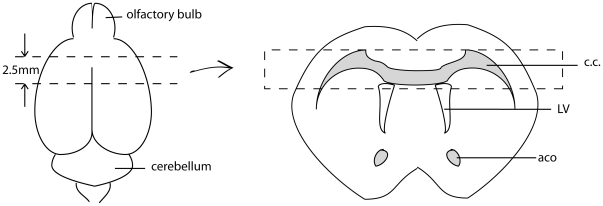
RNA sample sources. The illustration on the left represents the mouse brain. The top dashed line represents the first coronal cut, 5 mm away from the edge of olfactory bulb. The bottom dashed line represents the second coronal cut, 2.5 mm away from the first cut. The illustration on the right represents the brain section isolated on the left. The tissue within the dashed rectangle, containing mainly corpus callosum and part of the cortex, was used for RNA extraction. Abbreviations: CC – corpus callosum; LV – lateral ventricle; aca – anterior commissure.

All instruments used in the brain dissections were RNase-decontaminated by wrapping with RNaseZap® (Cat # 9780, Ambion). Mice were anesthetized by isoflurane inhalation and were quickly decapitated. The fresh brains were quickly removed and placed into a stainless-steel mouse-brain holder (Adult Mouse Brain Slicer Matrix, Cat # BSMAS005-1, ZIVIC Instruments). On the holder, a 2.5 mm thick coronal slab was made. The first coronal cut was 5 mm away from the edge of olfactory bulb, and the second cut was 2.5 mm (5 section slice intervals of the brain holder) away from the first cut. The selected brain structures (the corpus callosum and part of the cortex) were then dissected from the brain slab, using a stereomicroscope on ice (Fig. 6). Isolated tissues were quickly transferred into 2.0 ml RNase-free microtubes containing 0.6 ml of ice-cold TRIzol (Cat # 15596026, Invitrogen). Total RNA was extracted from each homogenized tissue sample (processed using Homogenizer Power Gen 125, Generator Flat 5×95, Fisher Scientific) using the phenol/chloroform extraction protocol provided by Invitrogen (TRIzol Reagent). Subsequently, the total RNA was purified using the RNeasy Mini Kit (Cat # 74004, QIAGEN) according to the manufacturer's protocol; DNA-decontamination treatment was included. The purified RNA samples were dissolved in RNase-free water and were stored at -80°C until processed.

### Real-time reverse transcription polymerase chain reaction

Total RNA was quantified using a spectrophotometer (NanoDrop, Cat # ND-1000, Thermo, Fisher Scientific), and 1 µg of total RNA was used to generate single-stranded cDNA. The concentrated total RNA samples were diluted to 0.1 µg/µl in at 10 µl volume. The diluted RNA samples were denatured at 70°C for 10 min and then added to the reverse transcription (RT) master-mix (Table 2). For RT, the reaction conditions were as follows: 25°C for 10 min, 42°C for 50 min, and 72°C for 15 min with a 0°C hold at the end.

**Table 2:** Primer pairs provided by SABiosciences™.

Symbol	Description	Band size	Ref pos	Exon	Cat#.
Gal	Galanin	139	607	6	PPM25148E
GalR1	Galanin receptor 1	151	1368	3	PPM04847A
GalR2	Galanin receptor 2	155	1261	2	PPM05170A
GAPDH	Glyceraldehyde-3-phosphate dehydrogenase	140	309	3	PPM02946E

The previously generated cDNA templates were diluted with 50 µl RNase-free water. For qPCR, the primer pairs were designed and generated by SABiosciences™, QIAGEN; the housekeeping gene GAPDH (Glyceraldehyde-3-phosphate dehydrogenase) was used as the internal quantitative control (Table 2). Each PCR consisted of 12.5 µl RT^2^ SYBR Green/ROX qPCR MasterMix (Cat # PA-012-24, SABiosciences, QIAGEN), 6.5 µl RNase-free water and 1 µl Primer Mix. PCRs for each gene of interest were run in triplicate using the StepOne™ Real-Time PCR System (Applied Biosystems™). The PCR cycling program was as follows: 95°C for 10 min and 40 cycles of 95°C for 15 s and 60°C for 1 min. The melt curve program was as follows: 95°C for 15 s, 60°C for 1 min, 65°C to 95°C at 2°C/min and 95°C for 12 s.

For validation of the primers, after each PCR cycling program a default melting program was run to make sure that disassociation curves for each pair of primers contained a single peak and the agorose gels of the amplified product revealed single band corresponding to the predictable amplicon length. To determine amplification efficiency a calibration curve was performed prior to the initiation of the experiments with excellent results.

### Data collection and processing

The body weight of the experimental animals was monitored twice a week. Body weight increases of the individual mice were calculated in terms of percentage of body weight increase compared to the initial body weight.

IHC staining slides were digitally captured using a ZEISS AxioImager A1 light microscope equipped with an AxioCam ICC3 digital camera, as well as with an OLYMPUS BH-2 light microscope equipped with an OLYMPUS Q-color5 digital camera. The software programs used for these two microscopes were AxioVision Rel. 4.8 version (Carl Zeiss) and ImagePro Plus 5.0 version (MediaCybernetics), respectively. The MBP-immunostaining revealed many fine fibers, which ran parallel to the cortex and were heavily condensed in the CC. We measured the optical density of the MBP-immunostaining according to NIH ImageJ protocols for quantitative comparison [42]. Because the intensity of the DAB color varied slightly between samples (due to natural variations between similarly treated samples), the optical density measurements were standardized for color intensity using the optical density of the aca (as demonstrated by the single-arrow in Fig. 2), an area where MBP staining was consistent, regardless of CPZ challenge. All of these data collection methods were also applied to PDGFR-α staining because proliferation of the positive cells in the experimental groups increased so dramatically that we could not accurately count them. Analysis of GST-π staining was based on actual counts by an experimental-blind personnel on whole fields of view.

### Statistical analysis

The statistic calculations and graphs were made using GraphPad Prism® version 5. Body weight data were analyzed using two-factor mix-measures analysis of variance (two-factor mix-measures ANOVA) assuming that genotype was the factor for independent grouping and that time elapsed was the other factor for repeated measures. All IHC data were analyzed using two-way independent ANOVA assuming that genotype was the factor for independent grouping and using groupings for different levels of experimental manipulation as another independent factor. All two-way ANOVA tests were accompanied by Bonferroni's multiple comparison tests. When considering variances caused by either factor (e.g., body weight changes within WT groups) one-factor ANOVA tests accompanied by Tukey's multiple comparison tests were applied. A p-value less than 0.05 (difference/effect) was considered to be significant.
